# Robust Incipient Fault Diagnosis of Rolling Element Bearings Under Small-Sample Conditions Using Refined Multiscale Rating Entropy

**DOI:** 10.3390/e28020240

**Published:** 2026-02-19

**Authors:** Shiqian Wu, Huiyu Liu, Liangliang Tao

**Affiliations:** 1 Shipbuilding Engineering Department, Jiangxi Polytechnic University, Jiujiang 332005, China; 2 Continuing Education College, Jiangxi Polytechnic University, Jiujiang 332005, China; 3 College of Information Engineering, Jiangxi Polytechnic University, Jiujiang 332005, China

**Keywords:** aero engine bearing, small sample fault diagnosis, refined time-shifted multiscale rating entropy

## Abstract

The operational reliability of aero-engines is critically dependent on the health of rolling element bearings, while incipient fault diagnosis remains particularly challenging under small-sample conditions. Although multiscale entropy methods are widely used for complexity analysis, conventional coarse-graining strategies suffer from severe information loss and unstable estimation when data are extremely limited. To address this, the primary objective of this study is to develop a robust diagnostic framework that ensures feature consistency and classification stability even with minimal training samples. Specifically, this paper proposes an integrated approach combining Refined Time-shifted Multiscale Rating Entropy (RTSMRaE) with an Animated Oat Optimization (AOO)-optimized Extreme Learning Machine (ELM). By introducing a refined time-shift operator and a dual-weight fusion mechanism, RTSMRaE effectively preserves transient impulsive features across multiple scales while suppressing stochastic fluctuations. Meanwhile, the AOO algorithm is employed to optimize the input weights and hidden biases of the ELM, alleviating performance instability caused by random initialization and improving generalization capability. Experimental validation on both laboratory-scale and real-world aviation bearing datasets demonstrates that the proposed RTSMRaE-AOO-ELM framework achieves a diagnostic accuracy of 99.47% with a standard deviation of ±0.48% using only five training samples per class. These results indicate that the proposed method offers superior diagnostic robustness and computational efficiency, providing a promising solution for intelligent condition monitoring in data-scarce industrial environments.

## 1. Introduction

Bearings serve essential functions in supporting rotating shafts and transmitting loads within mechanical systems, with their operational condition directly affecting the overall service life of equipment. In critical applications such as aero-engines, bearing failures can result in catastrophic consequences [1,2], thus necessitating effective early bearing fault diagnosis and accurate diagnosis. In the context of this study, early fault diagnosis is defined not merely by the microscopic physical dimensions of the defect, but by the signal-to-noise characteristics of the vibration response. It refers to the identification of incipient defects—such as minor spalling or indentations—that generate weak transient impulses heavily submerged in background noise and structural interferences. Detecting these weak signatures before they evolve into catastrophic failures is critical for predictive maintenance. However, early bearing fault signals exhibit nonlinear, non-stationary, and weak characteristics [3]. Additionally, fault samples are extremely limited in practical engineering applications, creating a typical small-sample problem that presents significant challenges to conventional bearing fault diagnosis methods [4].

Conventional bearing fault diagnosis methods can be broadly categorized into time-domain analysis, frequency-domain analysis, and time–frequency analysis. Time-domain analysis directly examines vibration signal waveforms and extracts statistical features such as mean, variance, and kurtosis [5]. This approach offers simplicity and ease of implementation. Frequency-domain analysis transforms original signals into the frequency domain using techniques such as Fourier transform, primarily analyzing the spectral structure of signals [6]. This method demonstrates high accuracy in identifying frequency components corresponding to known bearing fault characteristics. Time–frequency analysis employs methods such as wavelet transform and empirical mode decomposition to analyze the energy distribution of signals in the time–frequency domain [7,8]. This approach can handle non-stationary signals and captures localized information in both time and frequency domains. Although these methods perform well under specific conditions, they exhibit certain limitations in addressing early weak bearing faults and small-sample problems. Specifically, the linear statistical features used in time-domain and frequency-domain analysis cannot adequately characterize nonlinear fault information, while time–frequency analysis suffers from high computational complexity and the inherent trade-off between time and frequency resolution. To overcome the limitations of conventional methods, various advanced signal processing techniques have emerged in recent years. Variational mode decomposition (VMD) and its variants effectively suppress the mode-mixing issues inherent to empirical mode decomposition by iteratively optimizing the center frequencies and bandwidths of each mode [9,10,11,12]. Envelope spectrum analysis and resonance demodulation methods are specifically designed for bearing impact feature extraction, as they are capable of effectively amplifying fault signals and enhancing signal-to-noise ratios [13,14]. Sparse representation and compressed sensing techniques enable precise signal reconstruction while reducing sampling rates and demonstrate excellent denoising capabilities [15,16,17]. However, these methods still face challenges including sensitivity to parameter selection and high computational complexity, with their stability under small-sample conditions requiring further validation.

To address the critical challenge of small-sample fault diagnosis, researchers have proposed various solutions from both data and model perspectives. At the data level, Generative Adversarial Networks (GAN) and Variational Autoencoders (VAE) expand datasets by generating synthetic samples [18,19,20], while data augmentation combined with regularization techniques effectively prevents overfitting [21,22]. At the model level, few-shot learning, meta-learning, and transfer learning methods focus on improving model generalization capabilities under small-sample conditions [23,24]. More recently, advanced attention-based architectures, such as the Lightweight Multi-Scale and Multi-Dimensional Attention Transformer (LiMS-MFormer), have demonstrated robust fault diagnosis capabilities under complex operating conditions [25]. However, the reliability of these data-hungry deep learning models heavily depends on the availability of large-scale annotated datasets. In engineering scenarios with extreme data scarcity (e.g., 5-shot tasks), over-parameterized networks often suffer from instability and overfitting, making them unreliable for safety-critical applications. Meanwhile, deep learning approaches such as Convolutional Neural Networks (CNN) and Recurrent Neural Networks (RNN) demonstrate powerful feature extraction and classification capabilities under large-sample conditions [26,27]. However, these methods commonly suffer from complex network architectures, high computational costs, and unverified engineering stability, which limits their application in industrial engineering environments.

The above analysis demonstrates that in small-sample fault diagnosis, effective feature extraction based on physical mechanisms is often more critical than complex model architectures. This perspective is strongly supported by recent advances in physics-informed modeling; for instance, Cheng et al. [28] demonstrated how incorporating dynamics grounded in domain mechanisms is essential for shaping reliable surrogate models that align with physical realities. Inspired by this mechanism-guided paradigm, we argue that diagnostic features must fundamentally reflect the physical characteristics of faults (e.g., transient impulses) to ensure interpretability and robustness. On one hand, high-quality features can provide more stable diagnostic performance under limited sample conditions; on the other hand, features with clear physical meanings are more easily understood and accepted by engineering personnel, enhancing the engineering practicality of the methods. Therefore, exploring new feature extraction methods to better capture the essential characteristics of fault signals has become an important approach for solving small-sample fault diagnosis problems.

Entropy theory, as an important tool for measuring system complexity and uncertainty, demonstrates unique advantages in the fault diagnosis field [29]. Compared with conventional linear features, entropy features can effectively capture the dynamic characteristics of nonlinear signals and are more sensitive to early weak faults. From the development of single-scale entropy methods such as Approximate Entropy (AE), Sample Entropy (SE), and Permutation Entropy (PE) [30,31], entropy features have been increasingly applied in fault diagnosis. Recently, Li et al. proposed Rating Entropy [32], a novel complexity measure method that quantifies complexity by analyzing the swap count of signal permutation patterns. Compared with conventional entropy methods, Rating Entropy demonstrates significant advantages, including computational simplicity, strong noise resistance, and insensitivity to parameter variations, showing excellent performance in fault diagnosis. However, single-scale Rating Entropy still suffers from insufficient information comprehensiveness, and is unable to adequately reflect the complexity characteristics of signals across different time scales.

Consequently, multiscale entropy theory has been introduced into the fault diagnosis field [33]. Multiscale entropy methods such as Composite Multiscale Sample Entropy (CMSE), Refined Composite Multiscale Sample Entropy (RCMSE), Multiscale Permutation Entropy (MPE), and Multiscale Dispersion Entropy (MDE) generate signal sequences at different scales through coarse-graining processes, which are capable of revealing the dynamic characteristics of signals across multiple time scales and providing more comprehensive information for fault diagnosis [34,35,36,37]. However, multiscale methods face two critical issues: the information loss problem during the coarse-graining process, particularly at large scales where important information from the original signal may be masked by averaging operations; and the instability problem at large scales, where the reduction in effective data points leads to significant entropy value fluctuations, affecting diagnostic reliability. These problems become particularly prominent under small-sample conditions, severely limiting the practical application effectiveness of multiscale methods.

In addition to feature extraction, classifier performance is also a crucial factor for successful small-sample fault diagnosis. Extreme Learning Machine (ELM), as a fast single-hidden-layer feedforward neural network, possesses advantages such as rapid training speed and strong generalization capability, demonstrating excellent performance in small-sample classification tasks [38,39]. However, the random initialization strategy of ELM also introduces non-negligible challenges, where randomly selected input weights and biases may lead to poor network conditions, causing performance instability issues. Particularly under small-sample conditions, the randomness of network parameters may severely affect classification accuracy, resulting in significant variations in experimental results on the same dataset. To overcome this limitation, researchers have widely adopted metaheuristic optimization algorithms to optimize ELM network parameters, with methods such as Particle Swarm Optimization (PSO), Genetic Algorithm (GA), Whale Optimization Algorithm (WOA), and Grey Wolf Optimization (GWO) all achieving excellent results in ELM parameter optimization [40,41,42]. Among numerous optimization algorithms, Animated Oat Optimization Algorithm (AOO) emerges as a novel metaheuristic optimization method. AOO simulates the dynamic behavior of oats in wind and combines local search and global exploration mechanisms. It demonstrates outstanding advantages including fast convergence speed, strong global search capability, and simple parameter settings [43], effectively avoiding entrapment in local optima and providing a new effective approach for ELM parameter optimization, particularly achieving more stable and accurate classification performance under small-sample conditions.

Despite these advancements, a critical gap remains with regard to the reliability of diagnostic models under data-scarce conditions. Existing multiscale entropy methods suffer from statistical instability (i.e., fluctuating entropy values) when sample sizes are short, rendering the diagnostic outcome unreliable. The novelty of this research lies in bridging this specific reliability gap. By replacing the stochastic coarse-graining process with a deterministic time-shift strategy, the proposed RTSMRaE method ensures feature consistency, which is the cornerstone of reliable diagnosis. Coupled with the robust AOO-ELM classifier, this work provides a systematic solution to maximize reliability in data-restricted industrial environments.

The main contributions of this paper are summarized as follows:

(1) A novel feature extraction method termed Refined Time-shifted Multiscale Rating Entropy (RTSMRaE) is proposed. By replacing the traditional coarse-graining process with an intelligent time-shift strategy and a dual-weighting fusion mechanism, RTSMRaE effectively preserves the intrinsic temporal structure of fault signals and overcomes the statistical instability common in conventional multiscale entropy measures.

(2) An optimized classification paradigm, AOO-ELM, is developed to address the small-sample diagnostic challenge. The implementation of the Animated Oat Optimization (AOO) algorithm to fine-tune the input weights and biases of the Extreme Learning Machine (ELM) significantly mitigates the performance fluctuations caused by random parameter initialization, enhancing both diagnostic accuracy and stability.

(3) An integrated diagnostic framework is established by synergizing RTSMRaE feature extraction with the AOO-ELM classifier. This framework is specifically designed to handle the information sparsity inherent in limited datasets, providing a robust solution for early fault detection in complex mechanical systems.

(4) Extensive validation is conducted using simulated signals and two distinct experimental datasets, including the Harbin Institute of Technology (HIT) aviation bearing dataset and the Politecnico di Torino (PoliTO) dataset. The results demonstrate that the proposed method consistently achieves superior performance and exceptional generalization capability under extreme data scarcity compared to several state-of-the-art diagnostic approaches.

The remainder of this paper is organized as follows: Section 2 provides the mathematical foundation and algorithmic implementation of the proposed RTSMRaE method. Section 3 elaborates on the theoretical principles of the ELM classifier and the optimization mechanism of the AOO algorithm. Section 4 describes the complete procedure of the integrated bearing fault diagnosis framework. Section 5 presents the comprehensive experimental validation, including simulation studies, real-world aviation bearing datasets, and a detailed performance comparison with existing state-of-the-art methods. Section 6 provides an in-depth discussion on computational efficiency, industrial robustness, and the limitations of the proposed approach. Finally, the main findings and future research directions are summarized in Section 7.

## 2. Refined Time-Shifted Multiscale Rating Entropy

### 2.1. Rating Entropy Fundamentals

Rating Entropy (RaE), which is inherently distinct from probability-based entropy methods, quantifies complexity via the geometric structure of state space trajectories [32]. Unlike statistical measures that are sensitive to amplitude variations, RaE focuses on the ordinal patterns, offering superior robustness against environmental noise.

For a time series $x=\{x_{1},x_{2},\ldots ,x_{N}\}$, the phase space reconstruction is performed with embedding dimension *m* and delay $\tau_{d}=1$:

(1) $$X_{i}=\left\{x_{i},x_{i+1},\ldots ,x_{i+m-1}\right\},\;i=1,\ldots ,N-m+1$$

Each vector $X_{i}$ is mapped to a rank sequence $R_{i}$. The local complexity is defined by the minimum swap count $S_{i}$ required to sort $R_{i}$ into ascending order (equivalent to the inversion number in bubble sort). The global RaE is derived from the Shannon entropy of the swap count distribution p(s):

(2) $$\mathrm{RaE}=-\sum_{s=0}^{m(m-1)/2}p\left(s\right)\log_{2}p\left(s\right)$$

where *s* denotes the possible swap counts.

### 2.2. Refined Time-Shifted Multiscale Algorithm

Traditional multiscale methods utilize coarse-graining (averaging), which acts as a low-pass filter. While effective for long-term trends, this process inevitably obscures high-frequency fault transients, particularly in the large-scale factors. To address this information leakage, we propose the Refined Time-shifted Multiscale Rating Entropy (RTSMRaE).

#### 2.2.1. Time-Shift Operator

Instead of coarse-graining, a time-shift operator $T_{k}^{\tau }$ is introduced to generate τ distinct subsequences for a scale factor τ. This strategy preserves the pixel-level fidelity of the original signal:

(3) $$y_{k}^{\left(\tau \right)}=\left\{x_{k},x_{k+\tau },x_{k+2\tau },\ldots ,x_{k+j\tau }\right\},\;k=1,2,\ldots ,\tau$$

where j=⌊(N−k)/τ⌋. Let $RaE_{k}^{\left(\tau \right)}$ denote the Rating Entropy calculated for the *k*-th subsequence $y_{k}^{\left(\tau \right)}$.

It is crucial to highlight the fundamental difference between the proposed time-shift strategy and the coarse-graining process used in conventional refined methods (e.g., RCMSE). Traditional coarse-graining calculates the arithmetic mean of data points within a non-overlapping window, which mathematically functions as a linear low-pass filter. While this reduces noise variance, it inevitably smooths out the high-frequency transient impulses characteristic of incipient faults, altering the intrinsic ordinal patterns of the signal. In contrast, the time-shift operator employed in RTSMRaE generates subsequences through sliding start indices without amplitude averaging. This strategy creates a rigorous “pixel-level” reconstruction that preserves the raw fidelity of signal amplitudes and their rank sequences, ensuring that weak fault signatures are not masked by the smoothing effect, thereby providing a more physically truthful basis for complexity analysis.

#### 2.2.2. Dual-Weighting Fusion Mechanism

Since the generated subsequences vary in length and quality, a simple average may introduce bias. A dual-weighting strategy is constructed to synthesize the final entropy value:

(1) **Information Weight ($w_{L}$):** Longer subsequences carry more statistical significance. The length weight is defined as

(4) $$w_{L}^{\left(k\right)}=\frac{L_{k}}{\sum_{i=1}^{\tau }L_{i}}$$

where $L_{k}$ is the length of $y_{k}^{\left(\tau \right)}$.

(2) **Stability Weight ($w_{S}$):** To mitigate the impact of outliers caused by transient noise, a stability weight based on the median distance is introduced:

(5) $$w_{S}^{\left(k\right)}=\frac{\xi_{k}}{\sum_{i=1}^{\tau }\xi_{i}},\;\mathrm{where}\;\xi_{k}=\frac{1}{1+|RaE_{k}^{\left(\tau \right)}-\mathrm{med}\left({\mathrm{RaE}}^{\left(\tau \right)}\right)|}$$

The final RTSMRaE value is computed by fusing these components. An adaptive mechanism is further employed to handle extreme instability:

(6) $$\mathrm{RTSMRaE}\left(\tau \right)=\left\{\begin{array}{cc} \mathrm{med}({\mathrm{RaE}}^{\left(\tau \right)}) & \mathrm{if}\;\sigma >\theta \cdot \mu \\ \sum_{k=1}^{\tau }\left[\alpha w_{L}^{\left(k\right)}+\left(1-\alpha \right)w_{S}^{\left(k\right)}\right]\cdot RaE_{k}^{\left(\tau \right)} & \mathrm{otherwise} \end{array}\right.$$

where ${\mathrm{RaE}}^{\left(\tau \right)}=\left\{RaE_{1}^{\left(\tau \right)},\ldots ,RaE_{\tau }^{\left(\tau \right)}\right\}$. σ and μ denote the standard deviation and mean of the subspace entropy vector, respectively. The threshold θ is set to 0.5 to detect severe fluctuations, and the weighting coefficient α is set to 0.6 to prioritize information completeness while maintaining robustness.

The complete implementation procedure is formalized in Algorithm 1.
**Algorithm 1** Refined Time-shifted Multiscale Rating Entropy**Require:** Signal vector x, embedding dimension *m*, maximum scale $\tau_{\max}$**Ensure:** Multiscale feature vector $F\in R^{\tau_{\max}}$     1:**for** τ=1 to $\tau_{\max}$ **do**     2:    **Step 1: Time-shift Decomposition**     3:    Generate τ subsequences $Y^{\left(\tau \right)}=\left\{y_{1},\ldots ,y_{\tau }\right\}$ using Equation (3)     4:    **Step 2: Sub-scale Entropy Calculation**     5:    Compute entropy set $E=\{RaE\left(y_{k}\right)|y_{k}\in Y^{\left(\tau \right)}\}$     6:    Store lengths $L=\{|y_{k}||y_{k}\in Y^{\left(\tau \right)}\}$     7:    **Step 3: Adaptive Fusion**     8:    **if** σ(E)>0.5×μ(E) **then**     9:        $F_{\tau }\leftarrow \mathrm{median}\left(E\right)$   10:    **else**   11:        Calculate weights $w_{L}$ and $w_{S}$ via Equations (4) and (5)   12:        $w_{total}\leftarrow 0.6w_{L}+0.4w_{S}$   13:        $F_{\tau }\leftarrow \sum w_{total}\cdot E$   14:    **end if**   15:**end for**   16:**return** F

## 3. AOO-Optimized Extreme Learning Machine

### 3.1. Extreme Learning Machine Formalism

Extreme Learning Machine (ELM) is a single-hidden-layer feedforward network (SLFN) characterized by analytical weight determination rather than iterative gradient descent. Consider a dataset $D={\left\{\left(x_{i},t_{i}\right)\right\}}_{i=1}^{N}$, where $x_{i}\in R^{n}$ and $t_{i}\in R^{m}$. With *L* hidden nodes and activation function g(·), the ELM output is modeled as

(7) $$\sum_{j=1}^{L}\beta_{j}g\left(w_{j}\cdot x_{i}+b_{j}\right)=t_{i},\;i=1,\ldots ,N$$

where $w_{j}\in R^{n}$ and $b_{j}\in R$ denote the random input weights and biases, and $\beta_{j}\in R^{m}$ represents the output weights. Equation (7) can be compactly expressed in matrix form:

(8) Hβ=T

Here, $H\in R^{N\times L}$ is the hidden layer output matrix with entries $H_{ij}=g\left(w_{j}\cdot x_{i}+b_{j}\right)$, and $T\in R^{N\times m}$ is the target matrix. The optimal output weights $\hat{\beta }$ are obtained analytically via the Moore–Penrose pseudoinverse $H^{†}$:

(9) $$\hat{\beta }=H^{†}T$$

Despite the computational efficiency, the random initialization of parameters $\phi ={\left\{w_{j},b_{j}\right\}}_{j=1}^{L}$ often results in an ill-conditioned H, leading to unstable generalization in small-sample regimes.

### 3.2. Animated Oat Optimization (AOO)

To regularize the ELM parameter space, we employ the Animated Oat Optimization (AOO) algorithm [43]. AOO is a population-based metaheuristic that updates candidate solutions $X\in R^{d}$ through three distinct search mechanisms, simulating the dynamic behavior of oats.

Let $X_{i}^{t}$ denote the position of the *i*-th individual at iteration *t*, and let $X_{best}^{t}$ be the global optimum. The position update rules are defined as follows:

**(1) Local Exploitation (Gentle Breeze Strategy):** To refine solutions within a local neighborhood, the update follows:

(10) $$X_{i}^{t+1}=X_{i}^{t}+\alpha \cdot r_{1}⊙\left(X_{best}^{t}-X_{i}^{t}\right)$$

where α controls the exploitation intensity, $r_{1}\in {\left[0,1\right]}^{d}$ is a random vector, and ⊙ denotes the Hadamard product.

**(2) Exploration–Exploitation Balance (Moderate Wind Strategy):** To facilitate information exchange between individuals:

(11) $$X_{i}^{t+1}=X_{best}^{t}+\beta \cdot r_{2}⊙\left(X_{j}^{t}-X_{k}^{t}\right)$$

where indices j,k are randomly selected from the population, and β is a scaling factor.

**(3) Global Exploration (Strong Wind Strategy):** To escape local optima via large-scale displacement:

(12) $$X_{i}^{t+1}=X_{rand}^{t}+\gamma \cdot r_{3}⊙\left(X_{best}^{t}-X_{i}^{t}\right)$$

where $X_{rand}^{t}$ represents a randomly selected individual.

The selection of update strategies is governed by an adaptive probability mechanism that is dependent on the iteration progress, ensuring a dynamic trade-off between convergence speed and diversity.

### 3.3. AOO-ELM Optimization Framework

The stochastic instability of ELM is addressed by optimizing the input parameters ϕ. An individual in AOO encodes the vectorization of weights and biases:

(13) $$\theta ={\left[\mathrm{vec}{\left(W\right)}^{T},b^{T}\right]}^{T}\in R^{L(n+1)}$$

where $W=[w_{1},\ldots ,w_{L}]$ and $b=[b_{1},\ldots ,b_{L}]$.

The optimization problem is formulated to minimize the regularized training error. The fitness function J(θ) is defined as:

(14) $$J\left(\theta \right)=\sqrt{\frac{1}{N}{∥H\left(\theta \right)\hat{\beta }-T∥}_{F}^{2}}+\lambda {∥\hat{\beta }∥}_{2}$$

where ${∥\cdot ∥}_{F}$ is the Frobenius norm and λ is a regularization coefficient that is used to prevent overfitting. The first term minimizes empirical risk, while the second term enforces structural simplicity.

The integrated training procedure is detailed in Algorithm 2.
**Algorithm 2** AOO-Optimized ELM Training Strategy**Require:** Dataset D, Hidden nodes *L*, Population size $N_{pop}$, Max iterations $T_{max}$**Ensure:** Optimal ELM parameters $\theta^{*}$ and output weights ${\hat{\beta }}^{*}$     1:**Initialization:** Generate population $P^{0}=\left\{\theta_{1},\ldots ,\theta_{N_{pop}}\right\}$     2:**for** t=1 to $T_{max}$ **do**     3:    **for** i=1 to $N_{pop}$ **do**     4:        *Parameter Decoding:* Map $\theta_{i}$ to $W_{i},b_{i}$     5:        *ELM Solving:*     6:            Construct $H_{i}$ using $\left(W_{i},b_{i}\right)$     7:            Calculate ${\hat{\beta }}_{i}=H_{i}^{†}T$     8:        *Evaluation:* Compute fitness $J(\theta_{i})$ via Equation (14)     9:   **end for**   10:   Update global best $\theta_{best}^{t}=\arg\min_{\theta \in P}J\left(\theta \right)$   11:   **for** i=1 to $N_{pop}$ **do**   12:       Select strategy *S* ∈ {Equations (10)–(12)} based on adaptive probability   13:       Update position $\theta_{i}^{t+1}$ using strategy *S*   14:       Apply boundary constraints to $\theta_{i}^{t+1}$   15:   **end for**   16:**end for**   17:**return** $\theta^{*}=\theta_{best}^{T_{max}}$, ${\hat{\beta }}^{*}=H{\left(\theta^{*}\right)}^{†}T$

This integration effectively combines ELM’s computational efficiency with AOO’s powerful optimization capability, resulting in a classifier that maintains fast training characteristics while achieving enhanced accuracy and stability. This approach is particularly valuable for small-sample bearing fault diagnosis applications, where both computational efficiency and reliable performance are critical requirements.

## 4. Integrated Bearing Fault Diagnosis Framework

The proposed diagnostic framework establishes a systematic pipeline that maps raw vibration signals to specific fault categories. Mathematically, the diagnosis process can be modeled as a composite function Ψ:x↦y, integrating the feature extraction operator $F_{RTS}\left(\cdot \right)$ and the optimized classifier $C_{ELM}\left(\cdot \right)$.

The implementation procedure is systematically organized into three distinct phases, detailed as follows:

### 4.1. Phase 1: High-Fidelity Feature Space Construction

Let $S=\{x_{1},\ldots ,x_{M}\}$ denote the set of acquired raw vibration signals, where $x_{i}\in R^{N}$. Preprocessing is applied to normalize signal amplitudes. For each sample $x_{i}$, the RTSMRaE algorithm extracts a multi-scale complexity vector:

(15) $$f_{i}=F_{RTS}\left(x_{i}\right)={\left[\mathrm{RTSMRaE}\left(1\right),\ldots ,\mathrm{RTSMRaE}\left(\tau_{max}\right)\right]}^{T}\in R^{\tau_{max}}$$

This step transforms the high-dimensional, noisy time series into a compact, informative feature vector $f_{i}$, effectively reducing dimensionality while preserving fault-sensitive dynamics.

### 4.2. Phase 2: Classifier Optimization and Training

A training dataset $D_{train}={\left\{\left(f_{i},t_{i}\right)\right\}}_{i=1}^{M_{train}}$ is constructed, where $t_{i}$ is the one-hot encoded label vector. The AOO algorithm is employed to search for the optimal ELM structural parameters $\theta^{*}$ (input weights and biases) by minimizing the fitness function defined in Equation (14). Once $\theta^{*}$ is determined, the output weights ${\hat{\beta }}^{*}$ are analytically computed:

(16) $${\hat{\beta }}^{*}=H{\left(\theta^{*}\right)}^{†}T$$

This phase yields a trained classifier model parameterized by $\left\{\theta^{*},{\hat{\beta }}^{*}\right\}$.

### 4.3. Phase 3: Online Fault Diagnosis

For an incoming unknown test signal $x_{test}$, the diagnosis is executed via the forward propagation of the integrated model:

(17) $$o_{test}={\hat{\beta }}^{*}\cdot g\left(W^{*}x_{test}+b^{*}\right)$$

The final predicted fault class $\hat{y}$ is determined by the index of the maximum activation:

(18) $$\hat{y}=\arg\max_{c\in \{1,\ldots ,C\}}{\left(o_{test}\right)}_{c}$$

This structured framework ensures that the stochasticity of the input weights is constrained by the AOO optimization, guaranteeing consistent diagnostic performance.

## 5. Experimental Validation

To validate the effectiveness of the proposed RTSMRaE-AOO-ELM framework, comprehensive experiments are conducted using both simulated and real-world bearing fault datasets. The experimental validation aims to demonstrate three key aspects: the adaptability and superiority of the RTSMRaE method through simulation studies, the overall framework’s effectiveness using real aviation bearing datasets, and the method’s advantages through comparative analysis with existing approaches. The experimental design employs simulated signals for controlled evaluation of RTSMRaE performance under various conditions, and two real-world aviation bearing fault datasets from Harbin Institute of Technology (HIT) [44] and Politecnico di Torino [45] for practical validation. Additionally, comprehensive comparative experiments are conducted to evaluate different feature extraction methods, varying training sample sizes, and alternative fault diagnosis approaches. All experiments are implemented on a workstation equipped with Intel Core i7-12600KF processor (Intel Corp., Santa Clara, CA, USA), NVIDIA GeForce RTX 4060 graphics card (NVIDIA Corp., Santa Clara, CA, USA), and 64 GB RAM to ensure consistent computational performance.

### 5.1. Parameter Configuration and Optimization

To ensure the reproducibility of the proposed framework and facilitate its application in broader industrial scenarios, the key hyper-parameter settings are rigorously defined. A summary of the configuration for both the RTSMRaE feature extraction and the AOO-ELM classifier is presented in Table 1. The “Setting (Used)” column lists the specific values employed to generate the results in this study, while the “Recommended Range” offers guidance for adapting the method to different vibration datasets based on our sensitivity analysis.

### 5.2. RTSMRaE Algorithm Validation on Simulation Signals

Before applying the method to real-world bearing data, it is essential to theoretically validate the proposed RTSMRaE algorithm. We specifically focus on two critical properties for small-sample diagnosis: robustness to signal length and discriminative superiority over existing methods.

#### 5.2.1. Length Sensitivity Analysis

In practical engineering, acquiring long, high-quality signal sequences is often difficult. Therefore, the feature extractor must yield consistent results even with short data segments. To verify this, we generated synthetic 1/f noise signals with lengths varying from N=1024 to 5120.

**Figure 1:**
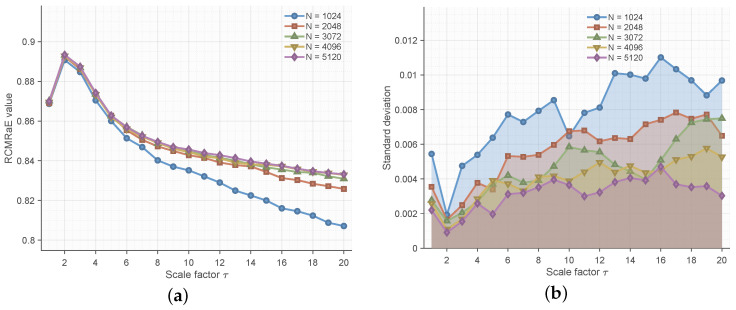
Length sensitivity analysis of RTSMRaE. (**a**) Mean entropy values remain consistent across different lengths; (**b**) Standard deviation stays at a negligible level.

Figure 1 illustrates the statistical performance of RTSMRaE across these lengths. **Consistency:** As shown in Figure 1a, the mean entropy values are remarkably stable. The curves for different data lengths almost perfectly overlap, particularly at scales τ>5. **Low Variance:** Figure 1b confirms that the standard deviation remains minimal (below 0.012) even for the shortest length (N=1024).

This result proves that RTSMRaE is insensitive to data length, making it ideal for limited-data scenarios where traditional methods might suffer from insufficient statistical samples.

#### 5.2.2. Comparative Superiority Analysis

To demonstrate the advanced performance of RTSMRaE, we conducted a comprehensive comparison against five state-of-the-art entropy methods: MRaE, RCMSlE, MSlE, RCMSE, and MSE. The goal was to distinguish between White Gaussian Noise (WGN) and 1/f noise, a classic benchmark for complexity measures.

**Figure 2:**
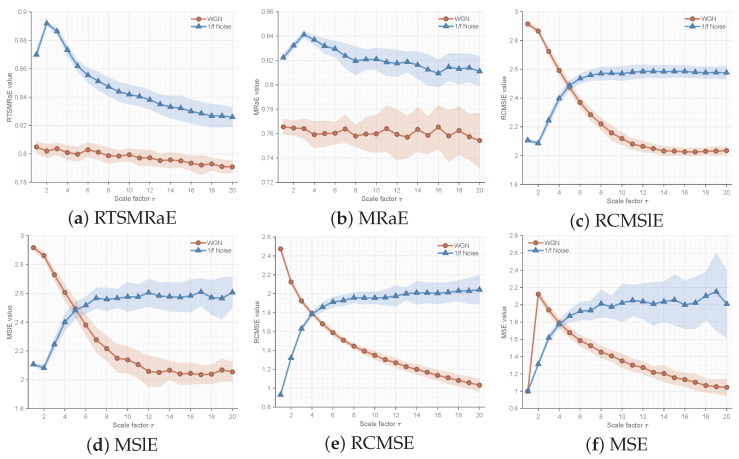
Comparison of entropy mean and standard deviation (shaded area) for WGN vs. 1/f noise. RTSMRaE shows the best separability and stability.

**(1) Analysis of Error Bands (Mean ± SD):** Figure 2 displays the entropy trends across 20 scales. RTSMRaE (Figure 2a) exhibits the clearest separation between the two noise types with the narrowest error bands (shaded areas). In contrast, traditional methods reveal significant limitations: **MSE & RCMSE (Figure 2e,f):** These methods show severe fluctuations and overlapping confidence intervals at higher scales (τ>10). This instability arises because the conventional coarse-graining process (averaging) causes information loss, especially when the effective data length shortens at large scales. **MSlE (Figure 2d):** While better than MSE, Slope Entropy still exhibits wider error bands compared to our method.

**Figure 3:**
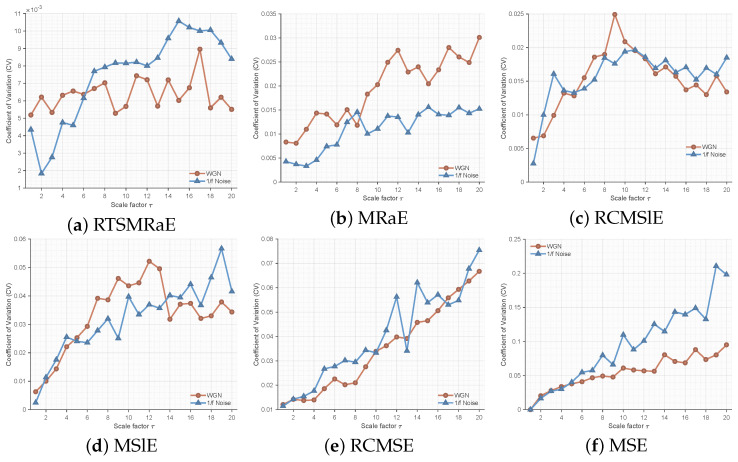
Coefficient of Variation (CV) comparison. Lower values indicate better stability. RTSMRaE achieves the most stable performance.

**(2) Analysis of Statistical Stability (CV):** To further quantify stability, we calculated the Coefficient of Variation (CV) for all methods, as shown in Figure 3. A lower CV indicates higher estimation reliability. The RTSMRaE method consistently maintains the lowest CV values (<0.001) across all scales. Notably, even compared to MRaE (Figure 3b), our refined time-shift strategy further suppresses variance. This quantitative evidence confirms that replacing “coarse-graining” with our “time-shift operator” significantly enhances the robustness of feature extraction.

In short, these simulation results prove that RTSMRaE is more stable and reliable than traditional entropy methods. It provides consistent features regardless of signal length and clearly distinguishes between different noise types, establishing a solid foundation for real-world fault diagnosis.

### 5.3. Real Aviation Bearing Dataset Validation

To evaluate the proposed framework in a mission-critical engineering context, we utilized the aviation bearing dataset from the Harbin Institute of Technology (HIT) [44]. Before discussing the results, it is essential to detail the technical condition of this unique experimental assembly.

**Figure 4:**
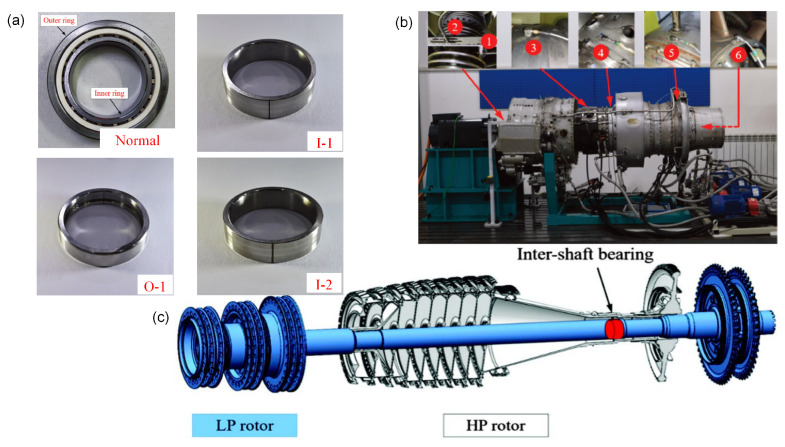
Technical configuration of the HIT experimental setup: (**a**) Photographs of the normal bearing and artificially induced faults; (**b**) the physical aero-engine test rig with labeled sensor locations (1–2: displacement, 3–6: acceleration); (**c**) 3D schematic of the dual-rotor architecture highlighting the inter-shaft bearing position.

The data was collected from a modified aero-engine test rig (Figure 4b) which preserves the core mechanical architecture of a turbofan engine. As illustrated in the dual-rotor schematic (Figure 4c), the critical component under analysis is the inter-shaft bearing located between the high-pressure (HP) and low-pressure (LP) rotors. This bearing is a cylindrical roller bearing with a pitch diameter of 55 mm and 15 rolling elements, subject to complex coupled excitations that differ significantly from standard single-rotor benches.

The vibration signature was captured using a comprehensive sensor array labeled in Figure 4b. Two eddy-current sensors (points 1–2, Kistler 8776A50M1) monitored the rotor displacement, while four acceleration sensors (points 3–6, Model K9000XL) were mounted orthogonally on the casing to capture the transmitted vibration. The data acquisition system recorded the response at a sampling rate of 25 kHz. Regarding fault simulation, Figure 4a displays the physical bearings with artificially induced defects. Wire-cutting discharge machining was employed to create precise faults on the outer and inner rings (0.5 mm width/depth) to simulate early-stage spalling damage. Although the seeded faults have specific dimensions (0.5 mm), the “early” nature of this diagnosis task is derived from the complex transmission path of the dual-rotor structure. Since the sensors are mounted on the external casing far from the inter-shaft bearing, the fault-induced impulses undergo severe attenuation and are modulated by the vibration of both high-speed and low-speed rotors. This results in a low signal-to-noise ratio scenario where the fault signature is barely discernible, effectively simulating the detection difficulty of incipient faults in real aero-engines.

The experiment covers four distinct bearing health states: Normal Condition (NOR), Inner Race Small Fault (IRS) with 0.5 mm depth, Inner Race Large Fault (IRL) with 1.0 mm depth, and Outer Race Large Fault (ORL) with 1.0 mm depth. Vibration signals were acquired at a high sampling rate of 25 kHz, while the low-pressure and high-pressure rotors operated at 3000 rpm and 3900 rpm, respectively. A critical challenge in aviation maintenance is the scarcity of fault data. To replicate this constraint, we adopted an extreme small-sample splitting strategy where only 5 samples per class were randomly selected for training, with the remaining samples used for testing. This 5-shot learning scenario imposes rigorous demands on the feature extraction capability.

In this study, the training set size was set to $N_{train}=5$ per class. This specific value was selected to adhere to the rigorous “5-shot” benchmark widely adopted in few-shot learning research [22,46]. While 10 or 20 samples are also common in small-sample studies, the 5-sample setting represents a scenario of extreme data scarcity, providing a more challenging stress test for the feature extraction capability of the proposed method. To mitigate the aleatoric uncertainty introduced by such a small sample size, the random selection process was repeated for 30 independent trials, ensuring that the reported performance reflects the method’s intrinsic stability rather than expanding on a fortunate data split.

#### 5.3.1. Feature Separability Analysis

High-quality features should minimize intra-class variance while maximizing inter-class distance. We compared RTSMRaE against five baseline methods (MRaE, RCMSlE, MSlE, RCMSE, and MSE) using both geometric visualization and numerical distribution analysis.

**Figure 5:**
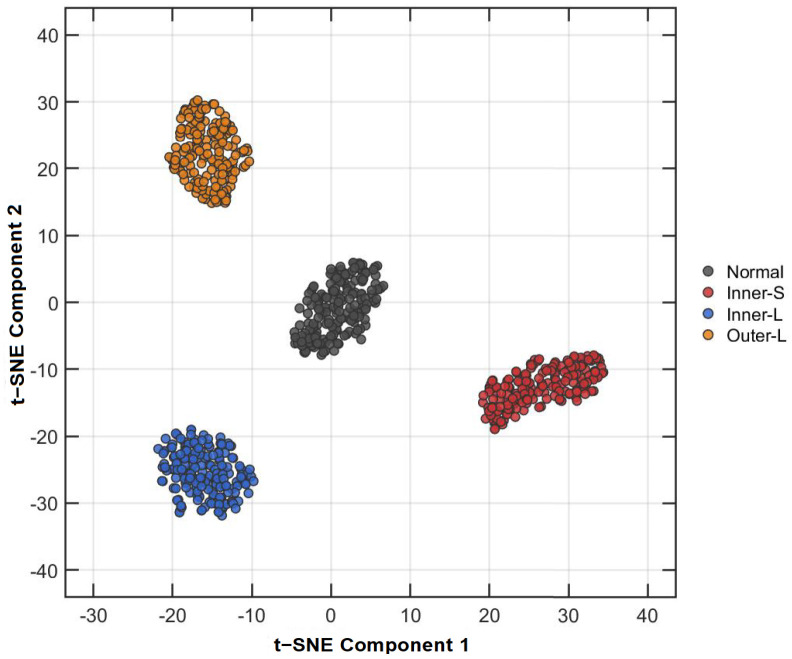
t-SNE visualization of RTSMRaE features. The distinct clusters demonstrate superior discriminative capability.

Figure 5 visualizes the feature distribution in a 2D space using t-SNE. The RTSMRaE features form four highly compact and distinct clusters, where the boundaries between different fault types are sharp and exhibit zero overlap. This geometric separation indicates that the proposed time-shift strategy successfully captures the unique signatures of different fault severities, distinguishing even between subtle inner race damage levels.

**Figure 6:**
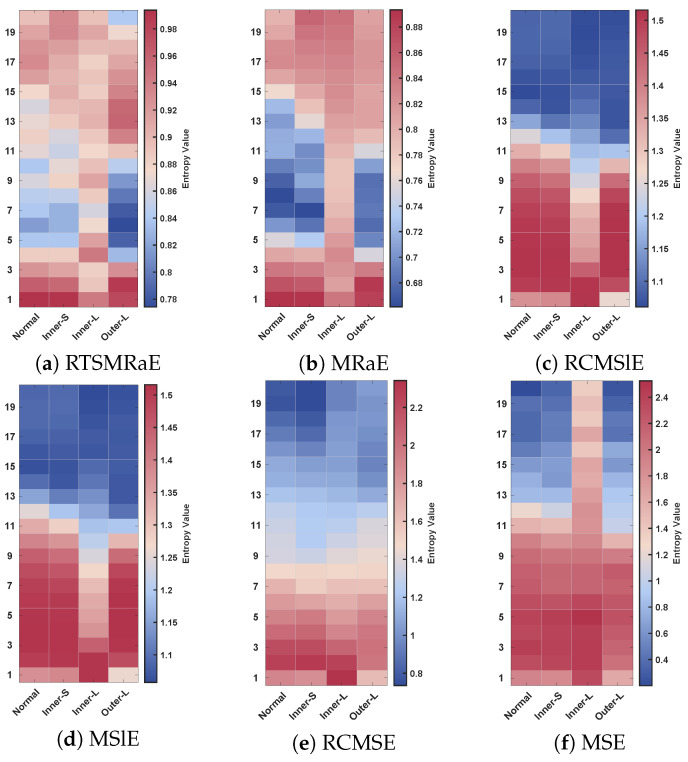
Entropy heatmaps across different scales. (**a**) RTSMRaE, (**b**) MRaE, (**c**) RCMSlE, (**d**) MSlE, (**e**) RCMSE, (**f**) MSE.

To further investigate the underlying consistency of these features, we examined the entropy values across scales, as shown in Figure 6. The RTSMRaE method (Figure 6a) exhibits the most robust patterns, where each fault type displays a unique color band that remains stable across all scales. This consistency is crucial for the classifier to learn reliable decision boundaries. In contrast, the entropy maps for MSE and RCMSE (Figure 6e,f) appear chaotic with mixed colors, failing to show clear differences between fault types. This confirms that conventional coarse-graining tends to destroy weak fault information in complex aviation signals. While MRaE (Figure 6b) performs better than sample entropy based methods, it still shows slight blurring at higher scales compared to RTSMRaE, validating the necessity of the refined dual-weighting improvement.

#### 5.3.2. Classification Performance Analysis

To verify the diagnostic reliability of the proposed framework, we evaluated the classification accuracy using the AOO-ELM classifier. A rigorous data-partitioning protocol was implemented to simulate extreme data scarcity: for each of the four health states, only 5 samples were randomly selected to construct the training set ($N_{train}=5$), while all remaining samples were retained for testing ($N_{test}$). Crucially, we employed a strictly non-overlapping sliding window approach to segment the raw vibration signals. This ensures that the training set and testing set share no common temporal data points, physically eliminating any possibility of data leakage. To eliminate the bias of random selection and ensure statistical reliability, this training–testing split was repeated for 30 independent trials. The feature vectors from all six entropy-based methods were processed under identical optimization conditions.

**Figure 7:**
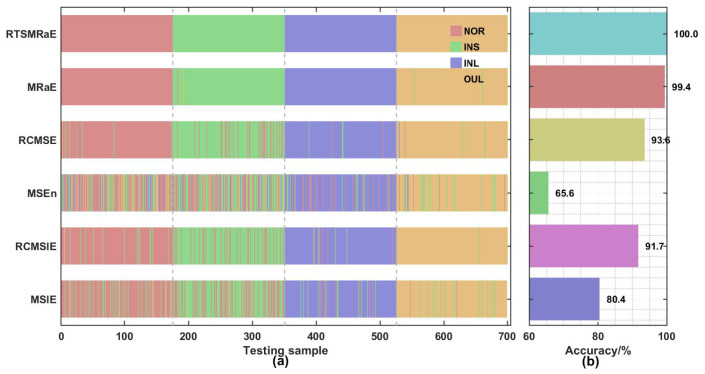
AOO-ELM classification performance comparison. (**a**) Prediction patterns for test samples showing fault category assignments; (**b**) quantitative accuracy comparison demonstrating the superiority of the proposed framework.

The assessment results are presented in Figure 7, which provides both sample-wise prediction patterns and overall accuracy metrics. As illustrated in Panel (a), the prediction output for RTSMRaE forms four clean, continuous color blocks without fragmentation. This visual continuity indicates that the classifier successfully established precise decision boundaries, achieving 100% diagnostic accuracy on the test set. Conversely, the prediction trajectories for MSE and MSlE exhibit frequent interruptions, representing misclassified samples where the model struggled to distinguish between the adjacent fault severities of the inner race.

The quantitative comparison in Panel (b) reveals a distinct performance hierarchy that corroborates our theoretical analysis. The RTSMRaE and MRaE methods consistently achieve top-tier performance, significantly outperforming the sample entropy and slope entropy baselines. This suggests that the underlying rank-based calculation of Rating Entropy is inherently more robust to the non-Gaussian characteristics of bearing fault signals. Moreover, a consistent trend is observed where refined composite methods (RCMSE, RCMSlE) outperform their standard counterparts (MSE, MSlE) by margins of approximately 2% to 5%. This validates the premise that suppressing statistical variance is essential for small-sample diagnosis. Most critically, the performance advantage of RTSMRaE over the standard MRaE confirms that the proposed time-shift strategy effectively eliminates the residual estimation uncertainty caused by traditional coarse-graining, ensuring maximum diagnostic fidelity.

#### 5.3.3. Small-Sample Adaptability Analysis

**Figure 8:**
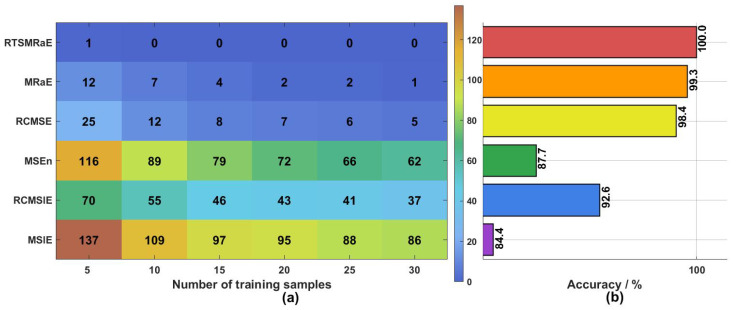
Small-sample performance analysis. (**a**) Classification error distribution heatmap: darker colors indicate higher error rates; (**b**) average accuracy curves showing the rapid convergence of RTSMRaE compared to baselines.

A pivotal metric for industrial applicability is the model’s ability to generalize from sparse training data. To evaluate this, we systematically varied the training set size from 5 to 30 samples per class, performing 30 independent trials for each configuration to ensure statistical reliability. The results, summarized in Figure 8, include both the cumulative error counts (Panel a) and average classification accuracy (Panel b).To provide a rigorous quantitative evaluation of the diagnostic stability, we have summarized the detailed accuracy rates and standard deviations across varying training sample sizes in Table 2. This table complements the visual trends shown in Figure 8.

The heatmap in Figure 8a reveals a striking contrast in stability under extreme data constraints. The proposed RTSMRaE method demonstrates exceptional robustness, maintaining near-perfect classification even when training data is scarce. Specifically, with only 5 training samples, RTSMRaE registered a negligible total of 1 misclassification across all trials. As the training set size increased to 10 or more, the method consistently achieved 100% accuracy. This indicates that the extracted features possess a high density of fault-related information, allowing the ELM classifier to converge to an optimal decision boundary rapidly.

The comparison with MRaE offers deeper insight into the algorithmic contribution. Although MRaE performed well overall, it exhibited noticeable instability at the 5-sample level, producing 12 misclassifications. Since both methods share the same Rating Entropy foundation, this performance gap isolates and validates the contribution of the time-shift strategy. Unlike the coarse-graining used in MRaE which effectively downsamples the signal, the time-shift operator preserves the full resolution of the original time series, thereby retaining subtle diagnostic clues that are critical when data is limited.

In contrast, traditional entropy methods struggled significantly in this regime. Sample entropy-based approaches (MSE and RCMSE) yielded the highest error rates, with MSE producing 116 misclassifications at the 5-sample point. This degradation is likely due to the inherent sensitivity of sample entropy to vector matching probabilities, which become statistically unreliable when the sequence length is short. While slope entropy methods (MSlE and RCMSlE) showed moderate improvement, they still lagged behind the Rating Entropy-based approaches. These results confirm that the proposed RTSMRaE-AOO-ELM framework successfully overcomes the dependency on large datasets, providing a viable solution for early fault diagnosis in data-sparse environments.

Overall, the validation on the HIT dataset shows that the proposed framework is highly effective when training data is extremely limited. By using only five samples per class, the method achieves nearly 100% accuracy and maintains clear separation between fault types, significantly outperforming standard entropy-based approaches.

### 5.4. Validation on Politecnico di Torino Bearing Dataset

To further substantiate the generalization capability and cross-domain robustness of the proposed framework, we extended the validation to the open-access Politecnico di Torino (PoliTO) dataset [45]. In contrast to the dual-rotor structure of the HIT dataset, this setup represents a high-speed spindle architecture.

**Figure 9:**
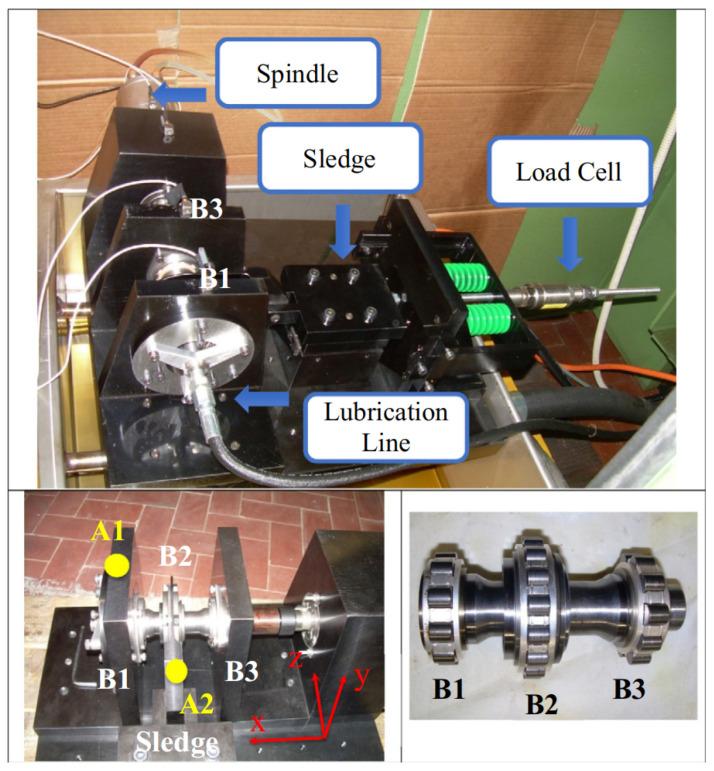
Schematic of the PoliTO high-speed bearing test rig, illustrating the spindle drive, bearing supports, and the spring-loaded radial loading mechanism.

The mechanical assembly, detailed in Figure 9, consists of a high-precision spindle driving a short shaft supported by three roller bearings. A distinguishing feature of this setup is the radial loading mechanism: the load is applied to the central bearing (B2) via a precision sledge mechanism connected to pre-loaded springs, monitored by a static load cell to ensure constant force application.

Vibration signals were acquired using triaxial IEPE accelerometers mounted on the rigid bearing supports (Sensitivity: 10 mV/(m/s^2^)). This setup generates high-frequency vibration data sampled at 51.2 kHz under varying rotational speeds up to 30,000 rpm. The faults were induced using a Rockwell indenter to create conical indentations on the inner ring and rollers, providing a rigorous benchmark for detecting weak fault signatures amidst heavy background noise. Similarly, the PoliTO dataset serves as a rigorous benchmark for early fault detection due to its high-speed operating conditions (up to 30,000 rpm). At such high rotational speeds, the background noise energy generated by the spindle and aerodynamic effects is substantial, often masking the transient signatures of the indentation faults. Identifying the fault status under these conditions requires extracting weak features from heavy noise, which aligns with the definition of incipient fault diagnosis in high-performance aeronautical components.

While the previous HIT dataset experiments confirmed the model’s effectiveness on discrete fault types, the PoliTO dataset introduces a higher dimension of complexity through systematic fault severity progression. This allows for a rigorous assessment of the algorithm’s sensitivity to subtle changes in signal dynamics, which is a prerequisite for precise prognostic health management in varying experimental environments.

The data originates from a high-speed aeronautical bearing test rig designed to simulate realistic flight conditions. For this study, we focused on the high-frequency operational condition of 12,000 rpm (200 Hz shaft frequency) sampled at 51.2 kHz, representing a challenging noise environment typical of aero-engines. The experimental design encompasses seven distinct health states: a normal baseline (NOR), three progressive inner ring faults (IF1, IF2, IF3) with increasing damage severity, two outer ring faults (OF1, OF2), and a ball fault (BF). This configuration constructs a demanding 7-class classification problem. The primary difficulty lies not merely in distinguishing between different component faults, but in discriminating between adjacent severity levels (e.g., IF1 versus IF2), where the vibration signatures exhibit high similarity and strong spectral aliasing.

For the validation protocol, we extracted 200 samples for each health state from the vertical acceleration channel, with each sample containing 2048 data points to ensure sufficient frequency resolution. This resulted in a comprehensive dataset of 1400 samples. Consistent with the HIT dataset experiments, we employed a non-overlapping sliding window approach to guarantee statistical independence between samples. This standardized setup serves to rigorously test whether the RTSMRaE features remain discriminative when identifying fine-grained fault evolutions under different mechanical structures and operational speeds.

#### 5.4.1. Entropy Pattern Analysis

To verify the discriminative capability of the features under this 7-class scenario, we performed a comparative analysis using geometric visualization (t-SNE) and numerical distribution assessment (Heatmaps). The primary goal is to determine whether the entropy features can successfully disentangle the coupled effects of fault location and damage severity.

**Figure 10:**
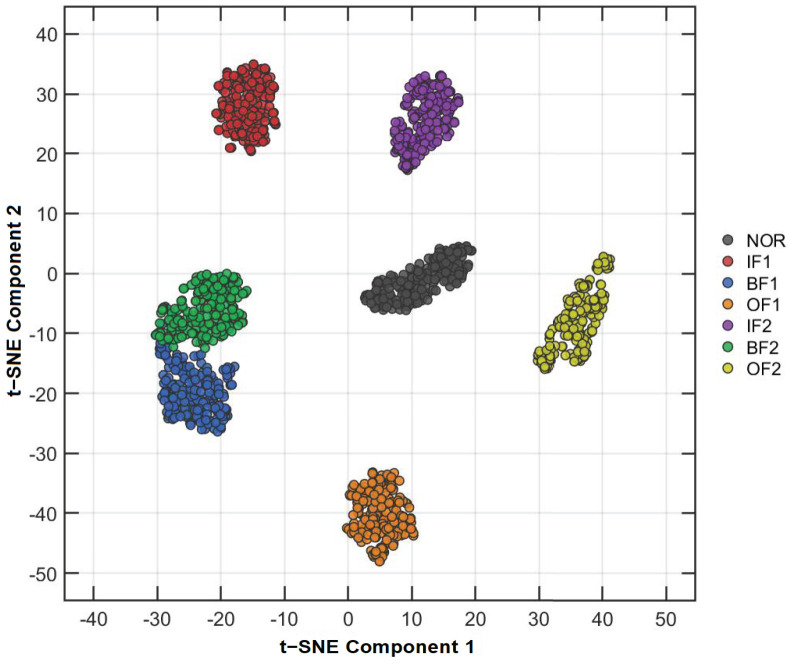
t-SNE visualization of RTSMRaE features on the PoliTO dataset. The clear separation between adjacent severity levels (e.g., IF1 vs. IF2) demonstrates high feature quality.

Figure 10 presents the t-SNE projection of the RTSMRaE feature vectors. The visualization reveals an exceptionally clear geometric structure: the seven health states map into seven compact, isolated clusters with large inter-class margins. Crucially, the method successfully resolves the “severity progression” challenge; the clusters for IF1, IF2, and IF3 are distinctly separated rather than merging into a continuous manifold. This indicates that the refined time-shift strategy preserves the subtle impulsive energy differences associated with varying defect sizes, preventing the feature aliasing often observed in traditional dimensionality reduction.

**Figure 11:**
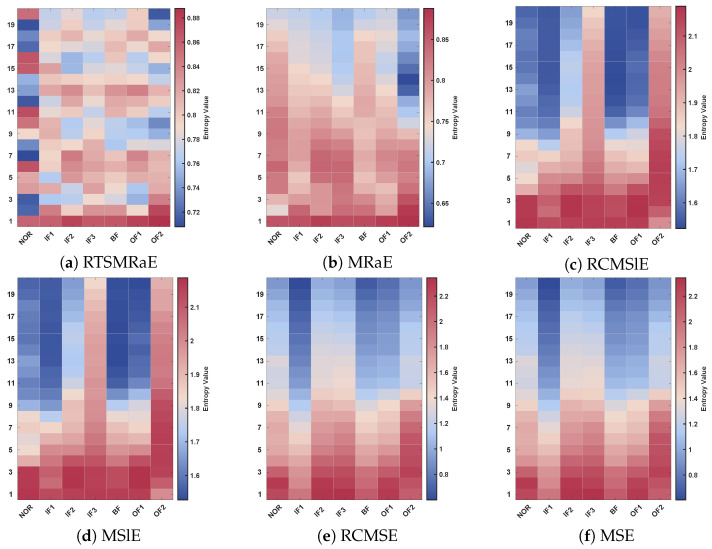
Multi-scale entropy heatmaps on the PoliTO dataset. (**a**) RTSMRaE, (**b**) MRaE, (**c**) RCMSlE, (**d**) MSlE, (**e**) RCMSE, (**f**) MSE.

The underlying mechanism for this separability is further elucidated by the multi-scale heatmaps in Figure 11. The RTSMRaE map (Panel a) displays a structured stratification effect, where the entropy magnitude correlates logically with the physical severity of the fault. A consistent gradient is observable as the inner ring fault progresses from incipient (IF1) to severe (IF3), and this pattern remains stable across all temporal scales. This monotonic relationship simplifies the decision boundary for the classifier.

In sharp contrast, the heatmaps for Sample Entropy (MSE, RCMSE) and Slope Entropy (MSlE, RCMSlE) exhibit significant disorder (Panels c–f). These methods fail to establish a stable correlation between entropy value and defect size, resulting in “color mixing” between the IF1 and IF2 categories. This instability stems from their reliance on amplitude thresholding, which is less sensitive to the ordinal changes caused by minor defect expansion. While the standard MRaE (Panel b) retains some structural clarity due to its rank-based nature, it exhibits noticeable blurring at higher scales (τ>10) compared to RTSMRaE. This comparison definitively proves that the proposed refinement strategy is essential for maintaining feature sharpness in high-complexity diagnostic tasks.

The distinct stratification observed in the RTSMRaE feature space provides a transparent link to the physical evolution of bearing degradation. In the early stage of a fault (e.g., IF1), the defect-induced impacts are weak and submerged in heavy background noise, resulting in a signal with high randomness and complexity. As the damage severity progresses to macroscopic spalling (IF2 to IF3), the fault mechanism shifts: the interaction between rolling elements and the defect generates high-energy, strictly periodic shock waves. These dominant impulses impose a deterministic “order” onto the time series, naturally reducing the ranking complexity. Crucially, the superiority of RTSMRaE lies in its ability to preserve this physical transition. Unlike traditional coarse-graining, which acts as a low-pass filter and blurs the sharp edges of these shock waves, the proposed time-shift strategy retains the fine-grained waveform structure. This ensures that the emergence of periodic fault impulses is accurately captured rather than being diluted back into noise, manifesting as the clear, monotonic gradient observed in the heatmaps.

#### 5.4.2. Classification Performance Analysis

To assess the cross-domain robustness of the proposed framework, we conducted classification experiments using the same 5-shot learning protocol (5 training samples per class) applied to the more complex 7-class PoliTO dataset. This scenario tests the model’s ability to maintain high precision when scaling from discrete fault types to fine-grained severity levels.

**Figure 12:**
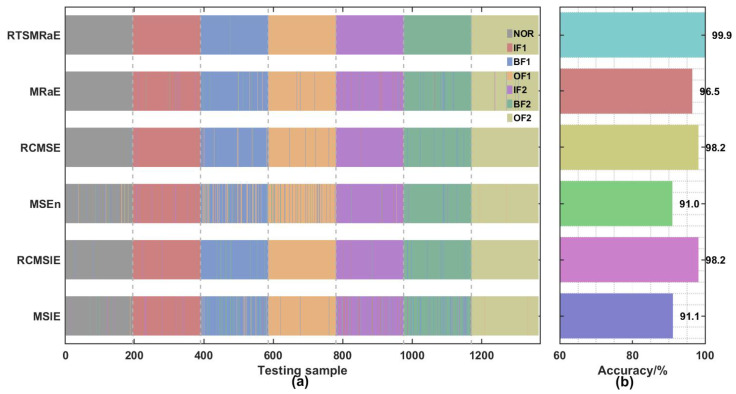
AOO-ELM classification performance on the PoliTO dataset. (**a**) Prediction patterns show clean separation between fault severities (e.g., IF1–IF3); (**b**) Accuracy comparison highlights the robustness of RTSMRaE in complex 7-class diagnosis.

The classification results, depicted in Figure 12, demonstrate the exceptional stability of the RTSMRaE-AOO-ELM framework. As shown in the prediction pattern (Panel a), the model achieves a near-perfect accuracy of 99.9%, with only negligible artifacts in the decision boundaries. This represents a minimal degradation of 0.1% compared to the HIT dataset baseline (100%), confirming that the method is invariant to changes in bearing geometry, rotational speed, and fault complexity. Notably, the prediction zones for the progressive inner ring faults (IF1, IF2, IF3) are clearly demarcated, indicating that the extracted features successfully encode the subtle energy increments associated with defect propagation.

The quantitative comparison in Panel (b) reveals a significant divergence in performance capability as the task complexity increases. While standard methods like MSE and MSlE struggled with the 7-class challenge (dropping to ≈91%), the refined composite methods (RCMSE, RCMSlE) maintained reasonable accuracy (≈98%).

Most critically, the comparison between RTSMRaE (99.9%) and its predecessor MRaE (96.5%) highlights the necessity of the proposed time-shift strategy. In the simpler HIT dataset, the performance gap between them was marginal; however, in this complex PoliTO scenario, the gap widens significantly. This suggests that while standard Rating Entropy is sufficient for identifying distinct fault locations, it lacks the resolution to distinguish between adjacent severity levels due to the smoothing effect of coarse-graining. By replacing coarse-graining with time-shifted sequencing, RTSMRaE preserves the high-frequency transient details required for fine-grained diagnosis, thereby ensuring superior performance even in challenging cross-domain applications.

To conclude, the PoliTO experiments confirm that the method is robust across different mechanical structures and speeds. It can accurately identify both fault locations and severity levels under high-speed conditions, proving its practical value for aero-engine health monitoring in varying environments.

### 5.5. Comparative Analysis with State-of-the-Art Methods

To strictly evaluate the proposed framework under data-scarce conditions, we conducted a rigorous comparative study using the PoliTO dataset. The experimental protocol was standardized such that for each of the seven bearing health states, only 5 samples were randomly selected for training, with the remaining 195 samples reserved for testing. This resulted in an extremely low training-to-testing ratio, imposing a severe test on the generalization capability of all diagnostic models.

#### 5.5.1. Baseline Methods Configuration

We selected five representative state-of-the-art methods as baselines, which we categorized into two mainstream technical paradigms: Deep Learning (DL) and Adaptive Decomposition (AD). For the DL paradigm, we compared against WSET-CNN-LSSVM [47] and FFT-CBAM-TCN [48]. The former transforms signals into Wavelet Synchrosqueezed time–frequency images fed into a CNN, while the latter utilizes an attention-based Temporal Convolutional Network to process spectral features. These methods represent the current trend of end-to-end diagnosis.

For the AD paradigm, we selected VMD-SABO-KELM [49], SSD-CMSDE-PSO-ELM [50], and FEEMD-CMSDE-PSO-ELM [51]. These approaches rely on signal decomposition techniques (VMD, SSD, FEEMD) to purify the signal before extracting entropy features. To ensure fair comparison, the specific parameters for all decomposition and optimization algorithms were set according to the best practices recommended in their respective original literature. In addition to the standard DL and AD paradigms, we further incorporated a Meta-Learning baseline to specifically address the few-shot challenge: the Prototypical Network (ProtoNet) [52]. Since open-source meta-learning implementations tailored for 1D bearing signals are scarce, we constructed a 1D-ProtoNet from scratch. The backbone utilizes a three-stage 1D-CNN (filters: 32-32-64) to map the 2048-point raw vibration signals into a 64-dimensional metric space. The model was trained under a 7-way 5-shot episodic framework using the Adam optimizer with a learning rate of 0.001, serving as a representative of metric-based few-shot learning methods.

#### 5.5.2. Performance Analysis and Discussion

The comparative results, detailed in Table 3, reveal a distinct performance hierarchy that exposes the fundamental limitations of competing technical routes under extreme data scarcity.

The comparison with deep learning and meta-learning baselines underscores the challenges of model complexity vs. data density. While the attention-based FFT-CBAM-TCN achieves a respectable 96.77% accuracy, standard deep models like WSET-CNN-LSSVM suffer from significant instability (86.67%). The root cause of this degradation is “model overfitting.” Standard deep architectures possess a massive number of parameters; when trained on only five samples, these highly flexible models tend to memorize the specific background noise and nuisance variations of the training subset rather than learning the intrinsic fault manifold. This leads to a model that fits the training data perfectly but fails to generalize to unseen test samples. The introduction of the 1D-ProtoNet yields a competitive accuracy of 93.60%, significantly outperforming the standard CNN. This confirms the advantage of the episodic training strategy in few-shot tasks. However, it still exhibits higher variance than our method, suggesting that generic metric-based meta-learning still requires a more diverse “support set” to form stable class prototypes in complex vibration environments.

Furthermore, the instability of adaptive decomposition methods highlights the sensitivity of signal-to-feature mapping. For methods like VMD-SABO-KELM, the performance fluctuations (±5.77%) are caused by “optimization bias.” In a 5-shot scenario, the meta-heuristic optimizers often converge to decomposition parameters (e.g., *K* and α) that are ideal for those five specific samples but are not representative of the broader data distribution, resulting in inconsistent features for the testing set. This issue is even more pronounced in FEEMD-based methods where mode mixing further distorts the entropy features.

Lastly, the failure of traditional entropy baselines like MSE and MSlE is rooted in “statistical instability.” Conventional coarse-graining averages data points, which effectively shrinks the sequence length. When the original samples are already short, shrinking them further makes the entropy calculation unreliable, as there are not enough data points left to provide a stable complexity estimate. In contrast, the proposed RTSMRaE ensures stability by using a deterministic time-shift strategy that maintains the full signal resolution. This provides our ELM-based classifier—an efficient analytical solver that is much harder to overfit—with high-fidelity features, ensuring consistent generalization even when constrained to a 5-shot regime.

## 6. Discussion

### 6.1. Computational Efficiency and Real-Time Feasibility

While the experimental results demonstrate superior diagnostic accuracy, the practical implementation of the proposed framework in direct industrial operations warrants further discussion regarding its computational feasibility. A critical distinction must be made between the model training phase and the real-time diagnostic phase. The parameter definition process, which involves Animated Oat Optimization (AOO) for tuning the input weights and biases, is computationally intensive. However, this optimization is executed entirely offline using historical training data. Once the optimal structural parameters $\theta^{*}$ and output weights ${\hat{\beta }}^{*}$ are determined, they are frozen as fixed constants for deployment.

During the direct operation (online monitoring), the system bypasses the iterative optimization process. The diagnostic workflow consists solely of extracting RTSMRaE features from the incoming vibration stream and performing a single forward propagation pass of the ELM network. Unlike deep learning models, which may require complex backpropagation or heavy convolution operations, the ELM inference relies exclusively on simple matrix multiplication. Consequently, the computational latency per sample is negligible, typically in the range of milliseconds. This “Offline Training—Online Diagnosis” architecture ensures that the high computational cost of the optimization algorithm does not compromise the real-time response capability required for aero-engine safety monitoring. Future work will focus on optimizing the feature extraction code to further minimize latency in embedded systems.

### 6.2. Industrial Robustness Under Variable and Noisy Conditions

The superior robustness observed across the HIT and PoliTO datasets can be attributed to the rank-based nature of the RTSMRaE feature extractor. Unlike traditional entropy variants that rely on amplitude thresholds, Rating Entropy focuses on the relative ordering of data points (swap counts). This ensures that the extracted features remain invariant to amplitude fluctuations typically caused by rotational speed shifts (ranging from 3000 to 12,000 RPM) or non-stationary industrial noise. This mechanism-guided behavior aligns with recent advancements in surrogate modeling [28], where aligning model behavior with physical realities is key to maintaining performance under complex operating conditions.

Crucially, the experimental results on both HIT and PoliTO datasets also substantiate the method’s immunity to background noise. As detailed in Section 5, the HIT dataset involves complex dual-rotor modulation, while the PoliTO dataset operates at ultra-high speeds (up to 30,000 rpm) with intense aerodynamic noise. These conditions represent realistic, high-noise industrial environments far more complex than simple Gaussian white noise. The fact that the proposed RTSMRaE-AOO-ELM framework maintains near-perfect accuracy (>99%) under these intrinsic low-SNR conditions confirms its ability to extract robust fault features from strong background noise, fulfilling the core requirement for incipient fault diagnosis.

### 6.3. Limitations and Parameter Sensitivity

Despite its advantages, the proposed framework has specific boundaries. As indicated in the parameter sensitivity analysis in Section 5, the performance is sensitive to extreme choices of the embedding dimension *m*. While a range of m∈[3,5] is effective, setting *m* too high leads to an exponential increase in the computational cost of feature extraction without significant gains in accuracy. Furthermore, as a meta-heuristic approach, the AOO algorithm does not mathematically guarantee a global optimum in every single run, though its stability has been demonstrated through 30 independent trials.

## 7. Conclusions

This work addresses the critical engineering challenge of diagnosing incipient bearing faults under conditions of extreme data scarcity. We proposed a hybrid diagnostic framework, RTSMRaE-AOO-ELM, which fundamentally rethinks the feature extraction paradigm for short time-series signals. By replacing the traditional coarse-graining averaging with a refined time-shift strategy, the RTSMRaE method successfully mitigates the information leakage inherent in multiscale entropy analysis, preserving high-frequency impulsive signatures that are typically smoothed out by conventional approaches.

The experimental validation on both the HIT and PoliTO datasets substantiates the robustness of this methodology. Most notably, under the severe constraint of 5-shot learning (5 training samples per class), the framework achieved a diagnostic accuracy of 99.47% with a minimal standard deviation of 0.48%. This performance significantly surpasses state-of-the-art deep learning and adaptive decomposition baselines, confirming that in small-sample regimes, preserving feature fidelity via rigorous signal processing is more effective than increasing model complexity. The integration of the AOO algorithm further regularizes the classifier, ensuring consistent generalization across varying operational speeds and fault severities.

In conclusion, the proposed framework offers a computationally efficient and theoretically sound solution for the health monitoring of aero-engine bearings, particularly in scenarios where fault data is expensive or hazardous to acquire. Future research will focus on extending this framework to variable-speed conditions and exploring physics-informed mechanisms to further enhance interpretability.

## Figures and Tables

**Table 1:** Summary of key parameter settings and recommended ranges for the RTSMRaE-AOO-ELM framework.

Module	Symbol	Parameter Description	Setting (Used)	Recommended Range
**RTSMRaE**	*m*	Embedding Dimension	3	[3,5]
	$\tau_{d}$	Time Delay	1	[1,3]
	$\tau_{max}$	Maximum Scale Factor	20	[10,30]
	α	Weighting Coefficient	0.6	[0.4,0.7]
**AOO-ELM**	$N_{pop}$	Population Size	30	[20,50]
	$T_{max}$	Max Iterations	100	[50,200]
	*L*	Hidden Layer Nodes	100	[50,500]
	g(·)	Activation Function	Sigmoid	Sigmoid/ReLU

**Table 2:** Detailed comparison of diagnostic accuracy (Mean ± Standard Deviation %) with varying training sample sizes ($N_{train}$ per class). The proposed RTSMRaE demonstrates superior stability and convergence speed (Bold is the indicator that shows the best effect).

Method	Number of Training Samples ($N_{\mathrm{train}}$)					
	5	10	15	20	25	30
**RTSMRaE**	**99.86 ± 0.34**	**99.99 ± 0.04**	**100.00 ± 0.00**	**100.00 ± 0.00**	**100.00 ± 0.00**	**100.00 ± 0.00**
MRaE	98.21 ± 1.19	98.94 ± 0.72	99.39 ± 0.35	99.77 ± 0.22	99.66 ± 0.28	99.79 ± 0.25
RCMSE	96.37 ± 1.96	98.31 ± 0.96	98.76 ± 0.53	98.89 ± 0.69	99.11 ± 0.46	99.18 ± 0.40
RCMSlE	90.03 ± 2.29	91.97 ± 1.03	92.96 ± 1.18	93.27 ± 0.93	93.41 ± 1.13	93.79 ± 1.15
MSEn	83.47 ± 2.75	86.93 ± 2.07	88.08 ± 1.48	88.72 ± 1.40	89.28 ± 1.34	89.68 ± 1.34
MSlE	80.46 ± 3.01	84.00 ± 1.48	85.37 ± 1.18	85.11 ± 1.10	85.83 ± 1.01	85.64 ± 1.18

**Table 3:** Performance Metrics Comparison on PoliTO Dataset (Mean ± Standard Deviation). Best results are marked in bold.

Method	Accuracy	Precision	Recall	F1-Score
**Proposed Framework**	**99.47% ± 0.48%**	**99.48% ± 0.47%**	**99.47% ± 0.48%**	**99.47% ± 0.49%**
FFT-CBAM-TCN	96.77% ± 1.84%	97.01% ± 1.63%	96.77% ± 1.84%	96.72% ± 1.91%
VMD-SABO-KELM	93.68% ± 5.77%	93.80% ± 5.20%	93.68% ± 5.77%	93.74% ± 5.49%
WSET-CNN-LSSVM	86.67% ± 6.41%	92.65% ± 2.27%	86.67% ± 6.41%	86.14% ± 7.07%
SSD-CMSDE-PSO-ELM	78.80% ± 2.64%	79.95% ± 2.45%	78.80% ± 2.64%	79.37% ± 2.52%
FEEMD-CMSDE-PSO-ELM	51.59% ± 1.20%	51.53% ± 7.26%	51.59% ± 1.20%	51.32% ± 3.78%
1D-ProtoNet	93.60% ± 2.66%	93.80% ± 2.72%	93.60% ± 2.66%	93.48% ± 2.75%

## Data Availability

The data used are unavailable due to privacy or ethical restrictions.
